# George Bridges

**Published:** 1892-02

**Authors:** 


### NECROLOGY.

### GEORGE BRIDGES.

### *IN MEMORIAM.*

*Resolutions of the Connecticut Veterinary Medical Association on the death of Dr. George Bridges.*

At a meeting of the Connecticut Veterinary Medical Association, held Tuesday, December 1st, 1891, the following preamble and resolutions were introduced by Dr. Thomas Bland, of Waterbury, and unanimously passed :

WHEREAS, Death—the great, the common fate of all—which spares neither high nor low, strong nor weak, learned nor simple, rich nor poor, young nor old, has called our brother, our associate, our friend, Dr. George Bridges, to rest from his life, and to lay down the cares, the burdens, the struggles and triumphs of earth. Therefore,

*Resolved*, By the Connecticut Veterinary Medical Association, that in the sad death of Dr. Bridges, this Association has lost a valued member, his profession an earnest worker, his associates a true-hearted and ardent friend. Thoroughly a master of his profession, he brought to it all his rare powers of judgment, aided and matured by experience. Of him his wide practice required marked ability, consummate tact, and a knowledge of the various branches of his profession at once thorough and complete. Yet, as a practitioner, he was ever equal to these demands ; and, as a scholar in veterinary medicine, he was exact and learned. It follows that he obtained readily and held easily a rank among the foremost of our honorable and humane fraternity.

Why, then, should we not, even in our sadness, delight to honor him in fitting eulogy. Aside from his marked professional abilities, Dr. Bridges had within himself that "touch of nature which makes all men kin." He was an honest, kind, genial, good-hearted and chivalric gentleman. There were no narrow by-ways, crooked lanes, or dark alleys in his life. Socially, he was the best and of the best. He scorned an unworthy even as he honored a noble act. How small the proportion of mankind of whom this much can truthfully be said. Despising shams, whether in the profession or out of it, Dr. Bridges had a rugged manhood peculiarly his own—a real nobility of character by Nature's patent given him—one far higher and more enduring than any which the potentates and princes of this earth can bestow. He was, indeed, one of Nature's noblemen, and to that high stature his whole life was squared.

"Take him for all in all,
Ne'er shall we look upon his like again."

*Resolved*, That this imperfect preamble and its accompanying resolutions be spread at length upon the records of this Association and a copy thereof transmitted to the wife and daughter of our dead brother and friend, which we beg them to accept, as a token of the esteem wherewith we hold the husband and the father, whose memory, in its many acts of kindness, deeds of worth and manliness of character, we shall, each and every one of us remember never to forget

